# Genotoxic effects of high dose rate X‐ray and low dose rate gamma radiation in Apc^Min/+^ mice

**DOI:** 10.1002/em.22121

**Published:** 2017-08-30

**Authors:** Anne Graupner, Dag M. Eide, Dag A. Brede, Michele Ellender, Elisabeth Lindbo Hansen, Deborah H. Oughton, Simon D. Bouffler, Gunnar Brunborg, Ann Karin Olsen

**Affiliations:** ^1^ Department of Molecular Biology Norwegian Institute of Public Health Oslo 0403 Norway; ^2^ Centre for Environmental Radioactivity (CoE CERAD) Ås 1432 Norway; ^3^ Department of Toxicology and Risk Assessment Norwegian Institute of Public Health Oslo 0403 Norway; ^4^ Department of Environmental Sciences Norwegian University of Life Sciences Ås 1432 Norway; ^5^ Radiation Effects Department Centre for Radiation, Chemical and Environmental Hazards, Public Health England Chilton Didcot OX11 0RQ England; ^6^ Department of Research Norwegian Radiation Protection Authority Østerås 1361 Norway

**Keywords:** chronic and acute irradiation, mutation, DNA lesions, micronuclei, blood cells

## Abstract

Risk estimates for radiation‐induced cancer in humans are based on epidemiological data largely drawn from the Japanese atomic bomb survivor studies, which received an acute high dose rate (HDR) ionising radiation. Limited knowledge exists about the effects of chronic low dose rate (LDR) exposure, particularly with respect to the application of the dose and dose rate effectiveness factor. As part of a study to investigate the development of colon cancer following chronic LDR vs. acute HDR radiation, this study presents the results of genotoxic effects in blood of exposed mice. CBAB6 F1 *Apc^+/+^* (wild type) and *Apc^Min/+^* mice were chronically exposed to estimated whole body absorbed doses of 1.7 or 3.2 Gy ^60^Co‐γ‐rays at a LDR (2.2 mGy h^−1^) or acutely exposed to 2.6 Gy HDR X‐rays (1.3 Gy min^−1^). Genotoxic endpoints assessed in blood included chromosomal damage (flow cytometry based micronuclei (MN) assay), mutation analyses (*Pig‐a* gene mutation assay), and levels of DNA lesions (Comet assay, single‐strand breaks (ssb), alkali labile sites (als), oxidized DNA bases). Ionising radiation (ca. 3 Gy) induced genotoxic effects dependent on the dose rate. Chromosomal aberrations (MN assay) increased 3‐ and 10‐fold after chronic LDR and acute HDR, respectively. Phenotypic mutation frequencies as well as DNA lesions (ssb/als) were modulated after acute HDR but not after chronic LDR. The *Apc^Min/+^* genotype did not influence the outcome in any of the investigated endpoints. The results herein will add to the scant data available on genotoxic effects following chronic LDR of ionising radiation. Environ. Mol. Mutagen. 58:560–569, 2017. © 2017 The Authors Environmental and Molecular Mutagenesis published by Wiley Periodicals, Inc. on behalf of Environmental Mutagen Society

## INTRODUCTION

The well‐characterised *Apc^Min/+^* mouse model of gastrointestinal tumorigenesis (*Apc*—adenomatous polyposis coli; *Min*—multiple intestinal neoplasia) (Luongo et al., 1994; Haines et al., 2000) has shown to develop gastrointestinal tumours after acute exposure to X‐rays (Luongo and Dove, 1996; van der Houven van Oordt et al., 1999; Ellender et al., 2005; Okamoto and Yonekawa, 2005; Ellender et al., 2006; Ellender et al., 2011). Less is known about the effects of chronic exposures to low dose rate (LDR) ionising radiation representing relevant exposure scenarios for radiation of humans. Furthermore, finding a blood‐based biomarker for the early effects of (chronic) radiation and thus possibly for gastro‐intestinal cancer has high relevance due to its transferable potential to humans and its minimal invasive sample acquisition.

Natural and man‐made sources of ionising radiation contribute to human exposures and are a recognized hazard for human health (Stannard, 1988). Risk estimates for radiation‐induced cancer in humans are based on epidemiological data largely drawn from the Japanese atomic bomb survivors. This group received external radiation at high dose rate (HDR) for a short period and at relatively high total doses, but show an increase in the prevalence of malignancies with increasing dose (Preston et al., 1987). Compilation of data from these studies together with a large number of other epidemiological and biological investigations, and animal studies at different dose rates, as well as high and low linear energy transfer (LET) radiation, have led to the conclusion that cancer risks from high dose and dose rate exposures were greater than the same dose given at lower dose rates (Ruhm et al., 2015). Because a linear extrapolation of cancer risks from high to low doses and dose rates was assumed to overestimate the risk at low doses, the ICRP and other international organisations have adopted the application of a reduction factor to estimate risks at low dose rates (LDR) and doses. This reduction factor has been termed as dose and dose‐rate effectiveness factor (DDREF). The use of the DDREF has been the subject of much debate in radiation protection, and recent publications have argued for revisions, or even the abolishment, in its application (SSK, 2014; Ruhm et al., 2015). Part of the controversy arises from the lack of information on effects in either animals or humans from chronic exposure to LDR radiation, particularly over different biological endpoints. According to UNSCEAR, LDR is defined as <6 mGy h^−1^ (UNSCEAR, 2010) and should be reported as such whatever the accumulated dose is (Wakeford and Tawn, 2010).

Long term studies in animals to study cancer and life shortening effects of high and low dose rates have already been performed since the 1970s (e.g., in beagle dogs (Thompson, 1989) and mice (Storer et al., 1979)). The combination of animal models and analysis of various molecular and cellular endpoints can provide important data for DDREF evaluation. Radiation is known to induce genotoxicity, which may be an indicator of cancer risk. Genotoxicity can be assessed for example by analysing chromosomal aberrations, phenotypic mutations, and DNA lesions. Chromosomal aberrations detected by micronuclei is an accepted marker for radiation exposure which was employed to evaluate effects of radiation on bank voles in the Chernobyl exclusion zone (Rodgers and Baker, 2000). DNA lesions followed by subsequent mutations have the potential to contribute to carcinogenesis. To our knowledge, only two other groups have applied the *Pig‐a* gene mutation assay as a tool to measure ionising radiation effects (Ohtani et al., 2012; Bhalli et al., 2013; Ohtani et al., 2014).

The present study compared the genotoxic effects of chronic LDR (2.2 mGy h^−1^) and acute high dose rate (HDR, 1.3 Gy min^−1^) exposures to low LET ionising radiation in the *Apc^Min/+^* mouse model. The presented results are part of a larger study with the objectives to (1) investigate whether the genotoxicity data in blood could be a marker for radiation induced tumour development, (2) calculate the risk of intestinal tumorigenesis for the same total dose (ca. 3 Gy) of acute (HDR) and chronic (LDR) radiation exposure effect, and (3) compare the DDREF across the different endpoints.

The hypothesis in this study was that different doses and dose rates of gamma rays differ in genotoxicity as assessed in blood with flow cytometry based micronuclei (MN) assay, *Pig‐a* gene mutation assay and DNA lesions in the Comet assay.

## MATERIALS AND METHODS

### Reagents

Lympholyte^®^‐Mammal cell separation reagent was from CedarLane, Burlington, ON, Canada. Anti‐PE MicroBeads, LS+ Positive Selection Columns and QuadroMACS™ Separator were from Miltenyi Biotec GmbH, Bergisch Gladbach, Germany. CountBright™ Absolute Counting Beads were from Invitrogen, Life Technologies™, Carlsbad, CA. Heat‐inactivated foetal bovine serum (FBS) was from PAA Laboratories, Pasching, Austria. Anticoagulant Solution, Buffered Salt Solution, Nucleic Acid Dye Solution (SYTO^®^13), Anti‐CD24‐PE and Anti‐CD61‐PE were from the MutaFlow^PLUS^ kit (mouse blood, tube‐based). This kit and the micronucleus analysis kit (*In Vivo* Mouse MicroFlow^PLUS^ kit) were from Litron Laboratories, Rochester, NY. *N*‐Nitroso‐*N*‐Ethylurea (ENU, cat. no. 3385) was purchased from Sigma–Aldrich Norway AS, Oslo, Norway. Low melting point agarose (NuSieve^®^GTG^®^Agarose) and Gelbond^®^ films were from Lonza, Rockland, ME. SYBR^®^Gold Nucleic Acid Gel Stain (10,000× concentrate in DMSO) was from Life Technologies™, Carlsbad, CA.

### Study Design

**Figure 1:**
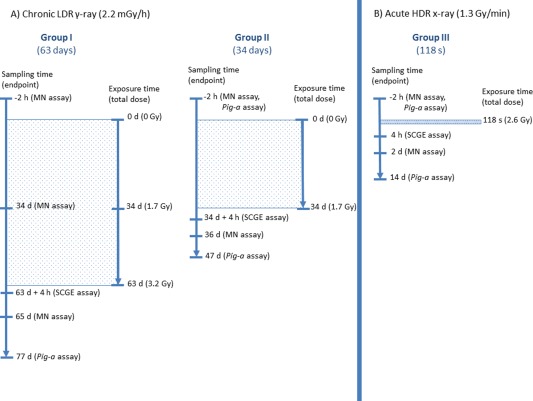
Overview

Mice were bred at Public Health England (PHE) and shipped to the Norwegian Institute of Public Health (NIPH) for chronic and acute exposure to ionising radiation (γ‐rays and X‐rays, respectively). The total whole body absorbed dose was approximately the same (3 Gy) while the whole body absorbed dose rate differed by a factor of more than 35,000 (2.2 mGy h^−1^ vs. 1.3 Gy min^−1^) (cf., Table 1 and Fig. 1). Different genotoxic endpoints were assessed in blood from the saphenous vein at NIPH (see below). Mice were then shipped back to PHE for analysis of gastrointestinal tumour counting (to be published elsewhere). While the whole study included female and male mice, only male mice were used to assess genotoxic endpoints and are presented in here.

**Table 1:** Characterisation of the Study Groups and Endpoints for the Herein Reported Mice (Males)

	Chronic LDR γ‐ray (2.2 mGy h^−1^)						Acute HDR X‐ray (1.3 Gy min^−1^)		
	Group I (63 days)			Group II (34 days)			Group III (118 s)		
		Number of animals per genotype^a^		Total absorbed body dose (Gy)	Number of animals per genotype			Number of animals per genotype	
	Total absorbed body dose (Gy)	*Apc^*+/+*^*	*Apc^*Min/+*^*		*Apc^*+/+*^*	*Apc^*Min/+*^*	Total absorbed body dose (Gy)	*Apc^*+/+*^*	*Apc^*Min/+*^*
Micronucleus assay (Fig. 1)	0, 1.7, 3.2	9	11	0, 1.7	9	11	0, 2.6	9	9
*Pig‐a* gene mutation assay (Fig. 2)	3.2	9	10	0, 1.7	9	11	0, 2.6	9	9
Single cell gel electrophoresis (Fig. 3)	3.2	8	8	0^b^, 1.7	8	8	0^b^, 2.6	9	9

Note: Blood samples were taken from the same mice prior, (during), and after irradiation.

a Genotype: wild type (*Apc^+/+^*) and mutant (*Apc^Min/+^*).

b Unexposed samples (0 Gy) are taken from a separate control group (i.e., from nonirradiated mice) on the same day.

### Radiation and Dosimetry

For dosimetry, please refer to Supporting Information Table S1. When designing the experiment it was aimed to expose the mice chronically and acutely with 3 Gy based on research on acute exposure to 2–5 Gy (Ellender et al., 2011) and previous experience with chronic exposure (Graupner et al., 2016). However, when using advanced models to determine total absorbed body dose (S1) it became clear that the actual total dose differs from the precalculated assumed doses (i.e., 3.2 Gy for chronic exposure and 2.6 Gy for acute exposure instead for precalculated 3 Gy).

#### Chronic Low Dose Rate Exposure

The chronic exposure was conducted at the LDR *Figaro* irradiation facility at the Norwegian University of Life Sciences (NMBU), Ås, Norway (Lind et al., FIGARO: Lessons learnt from constructing a low‐dose gamma irradiation facility (to be submitted)). Ninety mice (46 females, 44 males; not all of them were assigned to genotoxic endpoint testing) were continuously irradiated with γ‐rays from a ^60^Co source (450 GBq) at an air kerma rate of 2.3 mGy h^−1^. An Innovive rack with 5 × 5 cages was positioned at a distance of 650 cm from the center of the central cage to the source focus. The exposures took place for either 63 days to an estimated weighted average whole body absorbed dose of 3.15 Gy for the 26 females and 24 males in group I or for 34 days to an estimated weighted average whole body absorbed dose of 1.69 Gy for the 20 females and 20 males in group II (cf., Table 1). The continuous irradiation was interrupted on a daily basis for approximately two hours for animal care purposes. All cages were daily moved one position to the right to assure equal exposure throughout the whole irradiation period. Fifty unexposed control mice (22 females and 28 males; not all of them were assigned to genotoxic endpoint testing) were kept outside of the irradiation field but inside the *Figaro* facility behind lead shielding, under the same conditions as the exposed mice. The air kerma rate due to stray scatter behind the lead shielding was 5 µGy h^−1^.

#### Acute High Dose Rate Exposure

Thirty‐six mice (18 females and 18 males; not all of them were assigned to genotoxic endpoint testing) were acutely irradiated with X‐rays (1.52 Gy min^−1^ absorbed dose to water, 225 kV, 13 mA, 0.5 mm Cu‐filter, 52 cm distance to the source) from X‐RAD 225 (Precision X‐ray, North Branford) at NIPH receiving an estimated whole body absorbed dose of 2.6 Gy (cf., Table 1). Mice were placed in plastic tubes (50 mL with a breathing hole on the conic end) and thus immobilized for irradiation (time of irradiation: 118 s). One to three tubes were arranged in a circular pattern covering the field size with the conic ends facing the central field axis. Control mice (0 Gy) were also placed in plastic tubes (but kept outside of the radiation chamber) to simulate the stress situation.

### Animals

An inbred colony of C57BL/J *Apc^Min/+^* mice has been maintained at PHE since 1998 (Ellender et al., 2005). Female CBA/Ca mice (Envigo RMS (UK) Ltd, Bicester, UK) were mated with C57BL/6 *Apc^Min/+^* mice and the CBAB6 F1 progeny genotyped to identify *Apc^Min/+^* and *Apc^+/+^* (further referred to as “wild type”) mice.

Mice were acclimated for 1 week prior to irradiation start either in the irradiation facility *Figaro* or at the animal facility at NIPH. Mice were kept at a 12 h light/dark cycle and controlled temperature (20–24°C) and humidity (55% ± 10%) in air flow IVC racks (Innovive, San Diego, CA) according to the European convention 123, Appendix A (2006). At start of the irradiation mice were 4 to 8 weeks (chronic exposure) and 7 to 11 weeks (acute exposure) old, respectively. Four to five mice of the same sex were housed in one 100% PET disposable plastic cage with aspen bedding (Nestpack, Datesand, Manchester, UK). Water (public tap water) and feed (SDS RM1, Special Diet Services, Essex, UK), were given *ad libitum* to all mice.

A total of 212 mice (108 males and 104 females) were allocated to this study which will also include intestinal tumour counting at day 200 after the end of the exposure period (Ellender et al., data to be published elsewhere).

Two additional mice were treated with ENU (ip injection, 40 mg kg^−1^ bw on 3 consecutive days, total dose of 120 mg kg^−1^ bw) and used as positive controls in the *Pig‐a* gene mutation assay.

All procedures involving animals at PHE were carried out in accordance with the UK Animals (Scientific Procedures) Act 1986 and with guidance from the local AWERB at PHE. The experiments performed at NIPH/NMBU were in conformity with the laws and regulations for animal experiments in Norway and were approved by the Norwegian Animal Research Authority.

### Blood Sampling and Sampling Time Points

All genotoxic endpoints were analysed in whole blood of male CBAB6 F1 mice. Blood samples were taken from the saphenous vein using a 21‐G needle and a glass capillary tube (either coated with heparin (CAP‐MH‐75H) for the SCGE and *Pig‐a* gene mutation assay or EDTA (CAP‐MH‐75‐EDTA) for the MN assay (Bilbate, Daventry, UK). For the MN assay, 60 µL of free‐flowing blood was added to 350 µL anticoagulant (supplied with the MicroFlow^PLUS^, Litron Laboratories), mixed well, and kept at room temperature (RT). For the *Pig‐a* gene mutation assay 60 µL of free‐flowing blood was added to 100 µL anticoagulant (supplied with the MutaFlow^PLUS^, Litron Laboratories), mixed well, and kept at RT. For the SCGE assay 20–30 µl of free‐flowing blood was added to 100 µL anticoagulant (MutaFlow^PLUS^, Litron Laboratories), mixed well, and kept on ice. Further processing of all blood samples was performed within 2–6 h.

Blood samples for the MN and the *Pig‐a* gene mutation assay from all three groups were taken prior to radiation and 2 days (MN assay) or 2 weeks (*Pig‐a* gene mutation assay) after cessation of radiation. Blood for the MN assay from group I (3.2 Gy after 63 days) was also taken 32 days after the start of irradiation (at ∼1.7 Gy). Blood samples for the SCGE assay were taken 4 h after cessation of irradiation in compliance with the OECD TG 489 (OECD).

### Micronucleus (MN) Assay

Diluted blood samples (60 µL blood + 350 µL anticoagulant) were fixed in ultra‐cold pure methanol within 6 h and kept at −80°C for at least 3 days. Further processing and analysis of the samples were performed as described in the MicroFlow^PLUS^ (mouse blood) instruction manual (version140217) and elsewhere (Torous et al., 2003). Malaria‐infected erythrocytes served as a biological standard to calibrate the instrument. Approximately 20,000 CD71‐positive RETs per sample were acquired on a flow cytometer (BD LSRII, FACSDiva Software, BD Bioscience, San Jose, CA).

### *Pig‐a* Gene Mutation Assay

The *Pig‐a* gene mutation assay was performed as described in the MutaFlow^PLUS^ instruction manual (version140422). In short, red blood cells (RBC) and reticulocytes (RET, immature red blood cells) were leuko‐ and platelet‐depleted, washed with PBS and first incubated with anti‐CD24‐PE (labelling wild type (wt) RBC)/anti‐CD61‐PE (labelling platelets) and subsequently with anti‐PE magnetic particles (binding to the wild type‐RBCs and platelets). Afterwards a small fraction of each sample (10 µL) was taken and added to a Nucleic Acid Dye/Counting Bead Solution and incubated at RT for 10 min. This “pre‐column” sample provides information about the cell‐to‐bead‐ratio. The majority of the sample was transferred to a magnetic column (LS column, Miltenyi Biotec) to selectively remove CD24 positive cells and CD61 positive platelets. The eluate (enriched RBC^CD24−^ and RET^CD24−^) was concentrated, washed and subsequently incubated with 300 µL Nucleic Acid Dye/Counting Bead Solution at RT for 10 min. This so‐called “post‐column” sample gives information about the mutant‐to‐bead‐ratio. All pre‐ and post‐column samples were analysed using a flow cytometer (BD LSRII, FACSDiva Software, BD Bioscience, San Jose, CA) and their ratios are used to calculate the phenotypic mutant cell frequency as described previously for rats (Dertinger et al., 2011). The total numbers of RET and RBC equivalents are derived from the cell‐to‐bead ratios in the precolumn sample and the number of counting beads observed in the post‐column sample. On average this was 3 × 10^6^ RETs and 77 × 10^6^ RBCs per sample.

### Single Cell Gel Electrophoresis (SCGE)

A high throughput of the alkaline SCGE version (Gutzkow et al., 2013) was used, with minor modifications, and has been described in detail more recently (Graupner et al., 2014). Full blood samples (20–30 µL) were diluted with anticoagulant and analysed without further purification. Fifty randomly chosen comets per replicate (three technical replicates per mouse) were scored using 20× magnification with an Olympus BX51 microscope (light source: X‐Cite^®^ Series 120Q, Lumen Dynamics, Mississauga, ON, Canada; camera: BASLER, A312f‐VIS, Vision Technologies, Germany) and the Comet IV analysis software (Perceptive Instruments, Bury St. Edmunds, UK) was used to quantify the relative amount of fluorescing DNA in the comet tail vs. that of the whole comet (% tail DNA) as a measure of the level of DNA lesions in the individual cell. Data for net Fpg‐sensitive sites (Fpg‐ss) were obtained by subtracting % tail DNA of control samples (single strand breaks (ssb) and alkali labile sites (als)) from % tail DNA of samples treated with Fpg.

### Statistical Analysis

The data for the *Pig‐a* gene mutation assay was processed and calculated as described previously (Dertinger et al., 2011) using Microsoft Excel 2010. The raw data of the SCGE was processed using the Comet Assay Spreadsheet Generator Version 1.3.1 (Perceptive Instruments). The % TI (tail intensity, i.e. % tail DNA) of 50 comets were summarised as median (per gel) and 3 gels per animal (technical replicates) were summarised as mean as suggested by Bright et al. (Bright et al., 2011).

Further statistical analysis was performed using JMP Pro 12 (Statistical Analysis System Institute, Cary, NC). Data from the *Pig‐a* gene mutation assay and MN assay (chronic LDR exposure only) were log_10_‐transformed to achieve the best fit of normal distribution of residuals. An offset of 0.1 was added to values of RET^CD24−^ and RBC^CD24−^ prior to transformation.

The two factorial study design demanded analysis by a two‐way analysis of variance (ANOVA) in order to identify impacts of genotype, radiation, and interactions of both factors. Differences between groups (total doses) where further analysed by comparison of all pairs (Least Square MeansTukey HSD, applied for data from the MN assay and SCGE).

Data from the *chronic* exposure for the *Pig‐a* gene mutation assay was not normal distributed, neither after transformation. Therefore a non‐parametric test (Kruskal‐Wallis test) was applied to identify differences between the total doses (0, 1.7, 3.2 Gy) and genotypes (wild type or *Apc^Min/+^*).

Each group consisted of 8–11 mice, dependent on the method (cf., Table 1). For practical reasons there were no blood samplings of preirradiated mice for the *Pig‐a* gene mutation assay and SCGE of group I (cf., Table 1). Non‐irradiated blood samples from group II were therefore taken as control (i.e., 0 Gy). Data from the *chronic* exposure (i.e., group I and II) was pooled for further analysis in all three endpoints.

## RESULTS

**Figure 2:**
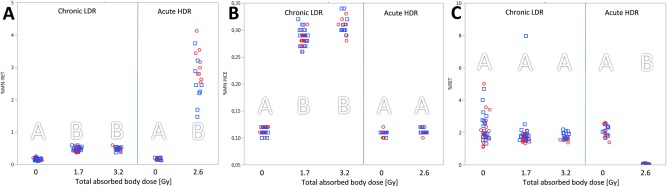
Micronuclei assay. Mice were either chronically exposed to gamma‐rays [2.2 mGy h^−1^, receiving a total absorbed body dose of 1.7 and 3.2 Gy (group I) or only 1.7 Gy (group II)] or acutely irradiated with X rays (1.3 Gy min^−1^, receiving a total absorbed body dose of 2.6 Gy (group III)); cf. Table I. Each data point represents one mouse: red open circles for CBAB6 F1 *Apc^+/+^* (i.e., wild type) and blue open boxes for *Apc^Min/+^*. The percentage of (**A**) micronucleated blood reticulocytes (% MN‐RET), (**B**) micronucleated normochromic erythrocytes (% MN‐NCE) and (**C**) relative reticulocyte population (% RET) are displayed. Same letters indicate that there is no significant difference between groups (*P* > 0.05); separate analysis for chronic and acute exposure.

**Figure 3:**
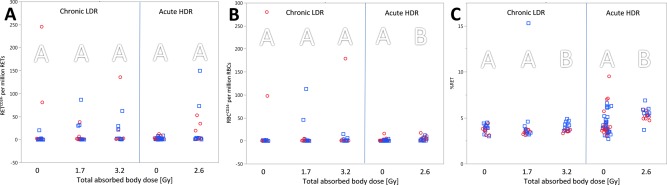
*Pig‐a* gene mutation assay. Refer to legend in Figure 2. Mutant phenotype frequencies of reticulocytes (RET^CD24−^, (**A**) and red blood cells (RBC^CD24−^, (**B**) and the relative reticulocyte population (% RET, **C**). Similar letters indicate that there is no significant difference between groups (*P* > 0.05); separate analysis for chronic and acute exposure.

**Figure 4:**
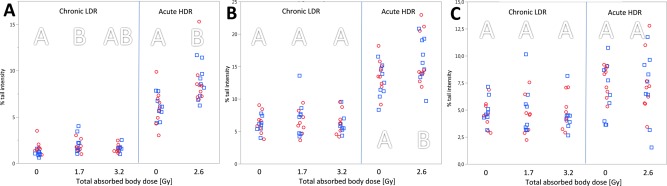
Single cell gel electrophoresis. Refer to legend in Figure 2. (**A**) Single strand breaks/alkali labile sites (ssb/als); (**B**) ssb/als and additional oxidised DNA lesions (assessed by Fpg); (**C**) Fpg‐sensitive sites (i.e., oxidised DNA lesions only) are displayed. Similar letters indicate that there is no significant difference between groups (*P* > 0.05); separate analysis for chronic and acute exposure.

Overall, a total dose of 1.7 Gy or 3.2 Gy of ionising radiation given chronically (gamma rays, 2.2 mGy h^−1^) or of 2.6 Gy given acutely (X rays, 1.3 Gy min^−1^) increased the frequency of chromosomal aberrations (MN assay) in peripheral mouse blood (Fig. 2). Data on acute irradiation also suggests an increased mutation frequency (Fig. 3) and increased level of DNA lesions (ssb/als, Fig. 4). The *Apc^Min/+^* genotype had no detectable impact on the assessed genotoxic endpoints.

In general, the SCGE assay reflects a recent induced effect (DNA damage, some hours), while the MN assay picks up effects after about 2 days and the *Pig‐a* gene mutation assay after some weeks. This has to be taken into consideration when looking at the presented results since the radiation duration varies from few seconds to several weeks.

In the following sections the results for each endpoint are described in more detail, starting with data from the chronic low dose rate (LDR) exposure (2.2 mGy h^−1^; groups I and II, cf., Table 1) and proceeding with data from the acute high dose rate (HDR) exposure (1.3 Gy min^−1^; group III, cf., Table 1). All data is summarised in the Supporting Information Table S2.

### Micronucleus (MN) Assay

Chronic LDR irradiation induced an approximately three‐fold increase of MN formation in immature (RET) and mature (NCE) blood cells (*P* < 0.001, two‐way ANOVA) (cf., Fig. 2A,B and Table 1, group I and II). There was no impact of the genotype (CBAB6 F1 wild type or *Apc^Min/+^*), nor any interactions between total dose of LDR irradiation (1.7 or 3.2 Gy) and genotype.

After acute exposure to X‐rays the relative amount of micronucleated reticulocytes (% MN‐RET) was more than ten times higher in blood of irradiated mice compared with non‐irradiated mice (*P* < 0.001, two‐way ANOVA; cf. Fig. 2A, group III). Mature micronucleated erythrocytes (% MN‐NCE) were increased by only 5% following radiation exposure (*P* = 0.07, two‐way ANOVA (cf., Fig. 2B, group III) and were within the range of unexposed mice from the chronic LDR experiment (i.e., groups I and II). The circulating reticulocyte population (% RET) was significantly reduced, from 2.08% to 0.05% (*P* < 0.001, two‐way ANOVA, cf. Fig. 2C, group III) which was also reflected by an increased analysis time at the flow cytometer.

### *Pig‐a* Gene Mutation Assay

Chronic exposure to gamma rays did not cause an increased mutation frequency in immature (reticulocytes) or mature red blood cells (RET^CD24−^ and RBC^CD24−^, respectively). This observation was independent of genotype (CBAB6 F1 wildtype or *Apc^Min/+^*) and the total absorbed body dose of 1.7 or 3.2 Gy given over a time period of 34 and 63 days, respectively (cf., Fig. 3A,B). The relative amount of total circulating immature blood cells (% RET) was slightly increased in *Apc^Min/+^* mice (18%) after receiving a total dose of 3.2 Gy (*P* = 0.002, Kruskal–Wallis test) (cf., Fig. 3C).

The mutation frequency of RETs (RET^CD24−^) in peripheral blood increased insignificantly after acute exposure to X‐rays (*P* = 0.06, two‐way ANOVA, cf., Fig. 3A, group III). On the other hand, the mutation frequency of mature blood cells (RBC^CD24−^) showed a 2.5‐fold increase following HDR irradiation (*P* < 0.002, two‐way ANOVA; cf., Fig. 2B, group III). Significant changes were also seen in the reticulocyte population (% RET) which was 1.5‐fold increased (*P* < 0.001, two‐way ANOVA; cf., Fig. 3C, group III). The genotype had no impact on the mutation frequency.

### Single Cell Gel Electrophoresis (SCGE)

Single strand breaks/alkali labile sites (ssb/als) and oxidised DNA lesions were measured by the SCGE without and with Fpg, respectively (Fig. 4A,B). There was a slightly increased level of ssb/als after chronic exposure to 1.7 Gy (from 1.4% TI to 2.0% TI, *P* = 0.050, two‐way ANOVA, Fig. 4A). The genotype had no significant impact on the level of DNA lesions.

Acute exposure with X‐rays caused a 30% increase in the levels of DNA lesions (ssb/als) from 6.1 to 8.8% TI (*P* < 0.001, two‐way ANOVA; cf., Fig. 4A). This trend was also visible when taking into account additional oxidised DNA lesions (*P* = 0.014, two‐way ANOVA; cf., Fig. 4B). However, the effect of radiation disappeared when taking into account the background level (i.e., calculating Fpg‐sensitive sites, Fig. 4C). The genotype had no significant impact on the level of DNA lesions.

## DISCUSSION

The overall aim of the whole project is to increase the knowledge on the development of colon cancer following chronic γ‐ray vs. acute X‐ray irradiation. This includes whether the incidence and severity of colon cancer development can be correlated with genotoxic responses observed in blood. In the herein presented part of the project, we aim to determine the genotoxic response of gamma radiation in blood at different dose rates. Both, the knowledge on genotoxicity in blood and on gastrointestinal cancer will contribute to evidence base relevant to the evaluation of DDREF. A series of different genotoxic endpoints were compared in the peripheral blood of CBAB6 F1 *Apc^Min/+^* and wild type mice receiving the same total dose (∼3 Gy) of ionising radiation (low LET). However, two different dose rates were applied, that is low dose rate (LDR, 2.2 mGy h^−1^) and high dose rate (HDR, 1.3 Gy min^−1^). Previous studies have addressed the influence of gamma dose rate on cancer in mice, demonstrating increased tumorigenic effectiveness of radiation exposure with increased dose rate (Ullrich and Storer, 1979). Moreover, the influence of dose rate on genotoxic and other endpoints has been extensively reviewed (Brooks et al., 2016).

In this study, both γ‐rays (chronic LDR) and X‐rays (acute HDR) were applied for irradiation. Even though they differ in their source and photon energy, both are low LET (linear energy transfer), i.e., a low amount of ionisation occurs along the track. Consequently, ionisation within the biological tissue is relatively homologous. However, it has been shown that 250 kVp X‐rays have a greater biological effect per cGy than γ‐rays from a ^60^Co source (Williams et al., 2010); that is up to 10% increase in the relative biological effectiveness (Spadinger and Palcic, 1992).

The genotoxic effects reported in this study have all been linked to ionising radiation, and the correlation with cancer risk. These include DNA lesions, gene mutations and chromosomal aberrations. Furthermore, mutagenicity is one of several hallmarks involved in cancer development (Hanahan and Weinberg, 2000; Hanahan and Weinberg, 2011). The methods used to investigate these endpoints are either established as OECD Technical Guidance (TG) protocols (OECD TG 474, MN assay and TG 489, SCGE assay) or under validation to become such protocols [*Pig‐a* gene mutation assay, reviewed in (Olsen et al., 2017)]. The three assays measure distinct but complementary facets of genotoxicity; the initial DNA damage levels with potential to become DNA mutations and the mutation analyses covering two classes of mutation. They are all performed in blood and thus easily applicable and in compliance with the three Rs (Replacement, Reduction, and Refinement). Furthermore, these endpoints have potential to be applied to assay human blood. The investigation of genotoxic endpoints in blood can elucidate very early adverse effects of radiation that may lead to cancer, in contrast to cancer diagnostics, which is highly resource demanding and has a lag time of several years.

Chronic (LDR) as well as acute (HDR) exposed mice showed a significantly increased level of MN in blood after receiving a total dose of ∼3 Gy. The level was more pronounced in the acutely exposed group (tenfold) than in the chronically exposed group (threefold)—compared with the unexposed groups (Fig. 2A). This observation suggests that MN formation is dose rate dependent. Data from the chronically exposed mice shows a saturation of MN formation in both RET and NCE at day 63 (Fig. 2A,B). This can partly be explained by the life span of erythrocytes in mice [38–58 days (Horky et al., 1978)] and partly by the lack of filtration of circulating MN‐containing erythrocytes (Abramsson‐Zetterberg et al., 1997). In a previous study with LDR we showed a similar response in the MN frequency in C57BL/6 mice (Graupner et al., 2016). The smaller observed fold‐increase in C57BL/6 compared to the CBAB6 F1 could be due to the higher radio resistant of the C57BL/6 strain (Grahn and Hamilton, 1957), and that the hybrid could be intermediate in sensitivity between C57BL/6 and CBA. Turner et al. (to our knowledge the only other study investigating dose rate dependent effects in mouse blood) showed no further increase at a total dose of 4.45 Gy (using a dose rate of 3.1 mGy min^−1^) (Turner et al., 2015). The herein applied MN assay is based on flow cytometry, counting >20,000 RETs (compared with a few thousand in the microscopic MN assay) thus giving much stronger and more reliable results.

In the present study, chronic LDR exposure did not induce an observable increase in phenotypic mutations in the *Pig‐a* gene mutation assay (Fig. 3). This is contrary to previous observations in our laboratory where we detected a three‐fold increase of the mutation frequency (RBC^CD24−^) after chronic exposure compared with nonirradiated mice [1.4 mGy h^−1^, 1.5 Gy total dose (Graupner et al., 2016)]. Acute exposure, on the other hand, appeared to induce mutations in red blood cells (RBC^CD24 − 1^). This is in line with studies by Ohtani et al. where mice received acute X‐ray exposure (0.52 Gy min^−1^, 1 and 2 Gy total dose)—the only ones to our knowledge using the *Pig‐a* gene mutation assay in mice to detect mutations caused by ionising radiation (Ohtani et al., 2012, 2014). Despite the lower dose rate and total dose, they observed an increased mutation frequency of both immature red blood cells [reticulocytes, RET^CD24−^, (Ohtani et al., 2012)] and mature red blood cells [RBC^CD24−^, (Ohtani et al., 2014)]. The herein acutely applied X‐ray dose (1.3 Gy min^−^, 2.6 Gy total dose) caused a significantly decreased reticulocyte population (% RET) 48 h after exposure in the MN assay (Fig. 2C). This was followed by a possible rebound in RET synthesis which would explain the increased % RET observed 2 weeks after acute exposure assessed by *Pig‐a* gene mutation assay (Fig. 3C). Such an increase has also been observed by others (Ohtani et al., 2014).

Unrepaired DNA lesions might result in mutations and possibly cancer. A link between high acute exposure to radiation and cancer (especially leukaemia) has been seen for atomic bomb survivors [reviewed in (Suzuki and Yamashita, 2012)]. In this study, DNA lesions were assessed by the SCGE. The chronic LDR exposure did cause a slight increase in the level of DNA lesions (ssb/als) after an accumulated dose of 1.7 Gy compared with the unexposed control group (Fig. 4A). A small amount of oxidised DNA lesions may have been induced through the handling of mice during acute exposure (i.e., placing mice in tubes): the unexposed mice from the acute exposure setting had a higher level of oxidised DNA lesions compared with unexposed mice from the chronic exposure setting (7.2% TI vs. 4.7% TI; Fig. 4C). The SCGE is sensitive enough to detect small differences, which has been shown for acute X‐ray (0–0.7 Gy) irradiated human lymphocytes (Gutzkow et al., 2013). The sampling time used in this study (4 h after irradiation stop) followed the one recommended in the OECD TG 489 for chemicals. The observation of such a small increase in ssb/als raises the question as whether an earlier time point would have been more reasonable with respect to the time of repair. Ssb/als in human lymphocytes are repaired within 30 min (Trzeciak et al., 2008). On the other hand, a 30% increase of the level of ssb/als was observed after acute exposure (Fig. 4A). This suggests that not the total dose but also the dose rate has a major impact on the type of DNA damage. Ionising radiation causes either direct or indirect effects. Direct effects occur when electrons hit DNA or other biomolecules, causing e.g. dsb. The formation of short‐lived hydroxyl radicals and other radicals (exogenous ROS) are summarised as the indirect effects of radiation. These radicals are able to attack the DNA and can cause DNA lesions, oxidised DNA and DNA clusters. To our surprise, no increased levels of oxidised DNA lesions were detected (Fpg‐ss), neither after acute nor chronic exposure (Fig. 4C). A study in breast cancer patients, however, showed that the level of urinary excreted 8‐oxo‐dG (marker for oxidised DNA lesions) can be used as marker for individual radiosensitivity (Haghdoost et al., 2001). Comparing the *control* groups in the SCGE from chronic and acute exposure with each other, a 17% increase in ssb/als was observed in the latter group. Unfortunately, we do not have any explanation for this. This observation may also be due to slight differences in housing or sampling conditions between the acute experiment and the chronic experiment, and it underlines the importance to use relevant controls in such experiments.

An important aspect of this study was to investigate genotoxic effects in a mouse model prone to develop gastrointestinal tumours within 200 days (*Apc^Min/+^*). Previous studies have found a correlation in T lymphocytes and fibroblasts *in vitro* to predict radiation‐induced damage in the small intestine (Kinashi et al., 1997). The results from our study show that low LET ionising radiation causes genotoxic effects dependent on the dose rate, i.e., LDR or HDR. These results were independent of the genotype, suggesting that the applied genotoxic endpoints assessed in blood might be unlikely to serve as specific markers for susceptibility to gastrointestinal cancer development.

The results of this study underline the importance of being aware of the biological consequences (genotoxic) at different dose rates, even though the total dose is the same. Both total dose and dose rate are essential information that needs to be stated (Wakeford and Tawn, 2010) as part of any experimental or epidemiological study. The present study demonstrates that the severity of certain genotoxic effects, namely chromosomal aberrations, is dependent of dose rate of ionising radiation (HDR vs. LDR). However, it is important to realize that effects on biomarkers that reflect the exposure may not correlate with increased risk of cancer (Brooks et al., 2003). Thus, the biomarker effects reported in this study will be compiled with tumour incidence data to evaluate the relationship between genotoxic endpoints and tumour development. Calculating the DDREF from a mechanistic perspective has been suggested (Ruhm et al., 2015). Thus, our MN data suggest a DDREF of at least three (i.e., 10‐fold increase (HDR)/3‐fold increase (LDR) of MN frequency = 3.3), the ratio of the slopes would be even higher. Recognising the limitation of a single genotoxicity assay, we will not contest the DDREF estimates of 1.5 (BEIR, 2006) to 2.0 (ICRP 2007) which are based on the risk of getting cancer. While reviews suggest that molecular and cellular measures of DDREF are greater than those seen for cancer rates, very few studies have attempted a direct comparison.

The current study stresses the importance of dose rate: the genotoxic impact of ionising radiation was not dependent on the applied total dose in the herein tested endpoints but on the dose rate. Incidences of chromosomal aberration, phenotypic mutation frequency and DNA lesions assessed in blood would seem unlikely to be applicable as direct markers for gastrointestinal cancer development, since no difference was seen between *Apc^Min/+^* and wild type mice. However, comparison of differences between tumour incidence rates following LDR and HDR irradiation should allow a more detailed identification of differences in the DDREF across different levels of biological endpoints.

## Supporting information

Supporting Information Tables.
