# Supplementary material for: Genotoxic effects of high dose rate X‐ray and low dose rate gamma radiation in ApcMin/+ mice
Source: Environ Mol Mutagen. 2017 Aug 30;58(8):560–9. doi: 10.1002/em.22121 (PMC5656900; doi:10.1002/em.22121)
Supplement: Supplementary file 1 — Supporting Information Tables. [file EM-58-560-s001.docx]

Supplementary tables

S1: Overview of the chronic and acute exposures of mice in groups I, II and III. For the chronic exposures, the exposure duration is the full duration of the experiments in days, and the beam-on time the actual exposure time in hours (taking into account breaks for the caretaking of mice etc.). In the chronic exposures, the center, minimum, maximum and weighted average air kerma rates are obtained from air kerma rates measured with nanoDots (Hansen and Hetland 2015), taking into account the time-dependent activity of the Co-60 source and the rotation of cages in the field. For the acute exposures, the measured dose quantity was the absorbed dose rate to water at a depth of 2 g/cm2 of water in a 21.0 cm x 21.0 cm x 11.5 cm large water phantom on the central field axis (Hansen et al. 2015). Estimated whole body absorbed dose rates are calculated from the measured dose quantities via conversion coefficients found from Monte Carlo simulations in Geant4 (Agostinelli et al. 2003; Allison et al. 2006). In the simulations, mice were modelled similar to the EULEP-EURADOS cuboid block PMMA mouse phantoms (Davies et al. 2000), with x,y,z-dimensions of 2.5 cm x 2.0 cm x 6.5 cm. The PMMA was replaced with ICRU four-component soft tissue. For the chronic exposure simulations, the beam was incident along x (hitting the side of a mouse) and z (hitting the front of a mouse) and results from the two exposures averaged. For the acute exposure simulations, the beam was incident along y (hitting the top of a mouse). The chronic exposure simulations took into account the source spectrum, the distance to the source focus and the geometry and composition of the phantoms. The acute exposure simulations additionally took into account backscatter from a substrate mice were resting on. The numeric value of the air kerma to whole body absorbed dose conversion coefficient for the chronic exposures was 0.932 ± 0.008. For the acute exposures, the numeric value for the absorbed dose to water to whole body absorbed dose conversion coefficient was 0.87 ± 0.04. The reported uncertainties are the combined standard uncertainties (BIPM et al. 2008), except in the case of the conversion coefficients, whose uncertainties are standard uncertainties arising solely from the statistics of repeated simulations.

|  |  | Group I | Group II | Group III |
| --- | --- | --- | --- | --- |
| Exposure | Facility | FIGARO | FIGARO | X-RAD 225 |
|  | Type | Chronic | Chronic | Acute |
|  | Beam | Co-60 gamma | Co-60 gamma | 225 kV x-ray |
|  |  |  |  |  |
|  | Start date | 22/01/2015 | 20/02/2015 | 05/05/2015 (males)  02/06/2015 (females) |
|  | Stop date | 26/03/2015 | 26/03/2015 | - |
|  | Duration | 63 d | 34 d | - |
|  | Beam-on time^[[1]](#footnote-1)^ | 1458.1 h | 788.6 h | 117.6 s |
| Air kerma rate | Min^[[2]](#footnote-2)^ | (2.09 ± 0.10) mGy/h | (2.08 ± 0.10) mGy/h | - |
|  | Max | (2.61 ± 0.13) mGy/h | (2.59 ± 0.13) mGy/h | - |
|  | Center^[[3]](#footnote-3)^ | (2.53 ± 0.18) mGy/h | (2.51 ± 0.18) mGy/h | - |
|  | Weighted average^[[4]](#footnote-4)^ | (2.31 ± 0.12) mGy/h | (2.30 ± 0.12) mGy/h | - |
| Air kerma | Min | (3.04 ± 0.15) Gy | (1.64 ± 0.08) Gy | - |
|  | Max | (3.80 ± 0.19) Gy | (2.04 ± 0.10) Gy | - |
|  | Center | (3.7 ± 0.3) Gy | (1.98 ± 0.14) Gy | - |
|  | Weighted average | (3.37 ± 0.17) Gy | (1.82 ± 0.09) Gy |  |
| Absorbed dose rate to water at 2 g/cm^2^ of water | Center^[[5]](#footnote-5)^ | - | - | (1.51 ± 0.03) Gy/min |
| Absorbed dose to water at 2 g/cm^2^ of water | Center | - | - | (2.96 ± 0.06) Gy |
| Estimated whole body absorbed dose rate^[[6]](#footnote-6)^ | Center | (2.35 ± 0.17) mGy/h | (2.34 ± 0.17) mGy/h | (1.32 ± 0.07) Gy/min |
|  | Weighted average | (2.16 ± 0.11) mGy/h | (2.15 ± 0.11) mGy/h | - |
| Estimated whole body absorbed dose | Center | (3.4 ± 0.2) Gy | (1.85 ± 0.13) Gy | (2.58 ± 0.13) Gy |
|  | Weighted average | (3.15 ± 0.16) Gy | (1.69 ± 0.09) Gy | - |

Agostinelli S, Allison J, Amako K, Apostolakis J, Araujo H, Arce P, Asai M, Axen D, Banerjee S, Barrand G, Behner F, Bellagamba L, Boudreau J, Broglia L, Brunengo A, Burkhardt H, Chauvie S, Chuma J, Chytracek R, Cooperman G, Cosmo G, Degtyarenko P, Dell'Acqua A, Depaola G, Dietrich D, Enami R, Feliciello A, Ferguson C, Fesefeldt H, Folger G, Foppiano F, Forti A, Garelli S, Giani S, Giannitrapani R, Gibin D, Gómez Cadenas JJ, González I, Gracia Abril G, Greeniaus G, Greiner W, Grichine V, Grossheim A, Guatelli S, Gumplinger P, Hamatsu R, Hashimoto K, Hasui H, Heikkinen A, Howard A, Ivanchenko V, Johnson A, Jones FW, Kallenbach J, Kanaya N, Kawabata M, Kawabata Y, Kawaguti M, Kelner S, Kent P, Kimura A, Kodama T, Kokoulin R, Kossov M, Kurashige H, Lamanna E, Lampén T, Lara V, Lefebure V, Lei F, Liendl M, Lockman W, Longo F, Magni S, Maire M, Medernach E, Minamimoto K, Mora de Freitas P, Morita Y, Murakami K, Nagamatu M, Nartallo R, Nieminen P, Nishimura T, Ohtsubo K, Okamura M, O'Neale S, Oohata Y, Paech K, Perl J, Pfeiffer A, Pia MG, Ranjard F, Rybin A, Sadilov S, Di Salvo E, Santin G, Sasaki T, Savvas N, Sawada Y, Scherer S, Sei S, Sirotenko V, Smith D, Starkov N, Stoecker H, Sulkimo J, Takahata M, Tanaka S, Tcherniaev E, Safai Tehrani E, Tropeano M, Truscott P, Uno H, Urban L, Urban P, Verderi M, Walkden A, Wander W, Weber H, Wellisch JP, Wenaus T, Williams DC, Wright D, Yamada T, Yoshida H, Zschiesche D. 2003. Geant4—a simulation toolkit. Nuclear Instruments and Methods in Physics Research Section A: Accelerators, Spectrometers, Detectors and Associated Equipment 506(3):250-303.

Allison J, Amako K, Apostolakis J, Araujo H, Dubois PA, Asai M, Barrand G, Capra R, Chauvie S, Chytracek R, Cirrone GAP, Cooperman G, Cosmo G, Cuttone G, Daquino GG, Donszelmann M, Dressel M, Folger G, Foppiano F, Generowicz J, Grichine V, Guatelli S, Gumplinger P, Heikkinen A, Hrivnacova I, Howard A, Incerti S, Ivanchenko V, Johnson T, Jones F, Koi T, Kokoulin R, Kossov M, Kurashige H, Lara V, Larsson S, Lei F, Link O, Longo F, Maire M, Mantero A, Mascialino B, McLaren I, Lorenzo PM, Minamimoto K, Murakami K, Nieminen P, Pandola L, Parlati S, Peralta L, Perl J, Pfeiffer A, Pia MG, Ribon A, Rodrigues P, Russo G, Sadilov S, Santin G, Sasaki T, Smith D, Starkov N, Tanaka S, Tcherniaev E, Tome B, Trindade A, Truscott P, Urban L, Verderi M, Walkden A, Wellisch JP, Williams DC, Wright D, Yoshida H. 2006. Geant4 developments and applications. Nuclear Science, IEEE Transactions on 53(1):270-278.

BIPM, IEC, IFCC, ILAC, ISO, IUPAC, IUPAP, OIML. 2008. Evaluation of measurement data - Guide to the expression of uncertainty in measurement (GUM 1995 with minor corrections).

Davies RW, Zoetelief J, Nikl I. 2000. Supplement IV. Animal phantoms for radiobiological dosimetry. Luxembourg, Belgium: European Commission.

Hansen EL, Hetland PO. 2015. Air kerma measurements with Landauer nanoDots in Cs-137 and Co-60 beams, Part I - SSDL reference exposures free in air. NRPA Technical Document Series 8.

Hansen EL, Ryste Hauge IH, Hetland PO, Bjerke H. 2015. Absorbed doses to water for x-ray dosimetry on a PXI X-RAD 225, Part I - Measurements. NRPA Technical Document Series 7.

S2: Detailed information on the measurement results for the three genotoxicity assays Micronucleus, Pig-a gene mutation and Single Cell Gel Electrophoresis. For more information refer to Table 1 and *Material and methods*.

| **Micronucleus assay** | | |  |  |  | Mean | | |  | Standard deviation | | |
| --- | --- | --- | --- | --- | --- | --- | --- | --- | --- | --- | --- | --- |
|  | Dose rate | Total dose | Genotype | N |  | %RET | %MN-RET | %MN-NCE |  | %RET | %MN-RET | %MN-NCE |
|  | acute (1.3 Gy/min) | 0 Gy | Apc^+/+^ | 9 | Group III | 2,1 | 0,2 | 0,1 |  | 0,4 | 0,0 | 0,0 |
|  |  |  | Apc^Min/+^ | 9 | Group III | 2,1 | 0,2 | 0,1 |  | 0,3 | 0,0 | 0,0 |
|  |  | 2.6 Gy | Apc^+/+^ | 9 | Group III | 0,0 | 3,1 | 0,1 |  | 0,0 | 0,5 | 0,0 |
|  |  |  | Apc^Min/+^ | 9 | Group III | 0,1 | 2,5 | 0,1 |  | 0,0 | 0,7 | 0,0 |
|  | chronic (2.2 mGy/h) | 0 Gy | Apc^+/+^ | 18 | Group I + II | 2,2 | 0,2 | 0,1 |  | 1,0 | 0,0 | 0,0 |
|  |  |  | Apc^Min/+^ | 22 | Group I + II | 2,3 | 0,2 | 0,1 |  | 0,9 | 0,0 | 0,0 |
|  |  | 1.7 Gy | Apc^+/+^ | 18 | Group I | 1,6 | 0,4 | 0,3 |  | 0,2 | 0,1 | 0,0 |
|  |  |  | Apc^Min/+^ | 22 | Group I | 2,1 | 0,5 | 0,3 |  | 1,4 | 0,1 | 0,0 |
|  |  | 3.2 Gy | Apc^+/+^ | 9 | Group I | 1,6 | 0,5 | 0,3 |  | 0,2 | 0,1 | 0,0 |
|  |  |  | Apc^Min/+^ | 11 | Group I | 1,8 | 0,5 | 0,3 |  | 0,2 | 0,1 | 0,0 |
|  |  |  |  |  |  |  |  |  |  |  |  |  |
| ***Pig-a* gene mutation assay** | | | |  |  | Mean | | |  | Standard deviation | | |
|  | Dose rate | Total dose | Genotype | N |  | %RET | RBC^CD24-^ x 10^-6^ | RET^CD24-^ x 10^-6^ |  | %RET | RBC^CD24-^ x 10^-6^ | RET^CD24-^ x 10^-6^ |
|  | chronic (2.2 mGy/h) | 0 Gy | Apc^+/+^ | 9 | Group II | 3,7 | 47,2 | 41,4 |  | 0,4 | 100,0 | 87,0 |
|  |  |  | Apc^Min/+^ | 11 | Group II | 3,9 | 0,6 | 2,7 |  | 0,5 | 0,8 | 5,9 |
|  |  | 1.7 Gy | Apc^+/+^ | 9 | Group II | 3,5 | 1,2 | 5,6 |  | 0,3 | 1,4 | 12,3 |
|  |  |  | Apc^Min/+^ | 11 | Group II | 4,7 | 15,2 | 14,1 |  | 3,5 | 35,2 | 27,0 |
|  |  | 3.2 Gy | Apc^+/+^ | 9 | Group I | 3,6 | 20,5 | 18,6 |  | 0,2 | 59,3 | 44,4 |
|  |  |  | Apc^Min/+^ | 10 | Group I | 4,2 | 2,9 | 12,4 |  | 0,5 | 4,7 | 20,4 |
|  | acute (1.3 Gy/min) | 0 Gy | Apc^+/+^ | 9 | Group III | 5,0 | 0,2 | 0,7 |  | 2,2 | 0,1 | 0,5 |
|  |  |  | Apc^Min/+^ | 9 | Group III | 4,6 | 0,3 | 1,3 |  | 1,0 | 0,3 | 1,2 |
|  |  | 2.6 Gy | Apc^+/+^ | 9 | Group III | 5,3 | 5,5 | 12,9 |  | 0,4 | 5,4 | 18,9 |
|  |  |  | Apc^Min/+^ | 9 | Group III | 5,5 | 3,8 | 26,8 |  | 0,8 | 4,0 | 51,7 |
|  |  |  |  |  |  |  |  |  |  |  |  |  |
| **Single Cell Gel Electrophoresis** | | | | |  | Mean | | |  | Standard deviation | | |
|  | Dose rate | Total dose | Genotype | N |  | %TI ssb/als | %TI ssb/als+Fpg-ss | %TI Fpg-ss |  | %TI ssb/als | %TI ssb/als+Fpg-ss | %TI Fpg-ss |
|  | chronic (2.2 mGy/h) | 0 Gy | Apc^+/+^ | 8 | Group II | 1,8 | 6,3 | 4,6 |  | 0,8 | 1,8 | 1,3 |
|  |  |  | Apc^Min/+^ | 8 | Group II | 1,1 | 6,1 | 5,0 |  | 0,3 | 1,2 | 1,3 |
|  |  | 1.7 Gy | Apc^+/+^ | 8 | Group II | 1,8 | 6,4 | 4,5 |  | 0,7 | 1,9 | 1,8 |
|  |  |  | Apc^Min/+^ | 8 | Group II | 2,2 | 7,3 | 5,1 |  | 1,1 | 3,0 | 2,4 |
|  |  | 3.2 Gy | Apc^+/+^ | 8 | Group I | 1,6 | 6,4 | 4,8 |  | 0,4 | 1,9 | 1,6 |
|  |  |  | Apc^Min/+^ | 8 | Group I | 1,7 | 6,2 | 4,5 |  | 0,4 | 1,6 | 1,6 |
|  | acute (1.3 Gy/min) | 0 Gy | Apc^+/+^ | 9 | Group III | 6,1 | 13,7 | 7,7 |  | 2,0 | 2,6 | 1,4 |
|  |  |  | Apc^Min/+^ | 9 | Group III | 6,1 | 12,7 | 6,6 |  | 1,3 | 2,6 | 2,6 |
|  |  | 2.6 Gy | Apc^+/+^ | 9 | Group III | 8,8 | 16,2 | 7,4 |  | 2,6 | 4,1 | 2,9 |
|  |  |  | Apc^Min/+^ | 9 | Group III | 8,8 | 15,9 | 7,1 |  | 1,9 | 3,5 | 3,2 |

1. The uncertainty on the beam-on exposure times are negligible compared with other uncertainties. [↑](#footnote-ref-1)
2. The combined relative standard uncertainty on the air kerma rates at fixed locations in the field measured with nanoDots is 5%. [↑](#footnote-ref-2)
3. The combined relative standard uncertainty on the air kerma rate in the center of the central cage is 7% because this value was interpolated between two measurements. [↑](#footnote-ref-3)
4. The combined relative standard uncertainty on the weighted average air kerma rate is set to 5% based on measurements where nanoDots followed cage rotations in the field. [↑](#footnote-ref-4)
5. The combined relative standard uncertainty on the absorbed dose rate to water at the reference depth on the central field axis in the water phantom is 2%. [↑](#footnote-ref-5)
6. The table reports estimated whole body absorbed dose rates based on the multiplication of the measured values with dose conversion coefficients. Therefore, the combined relative standard uncertainty on the estimated whole body absorbed dose rates results from uncertainties on the measured quantities and from the statistics of repeated simulations in the Monte Carlo simulations. [↑](#footnote-ref-6)
